# Gastric Adenocarcinoma after Gastric Bypass for Morbid Obesity: A Case Report and Review of the Literature

**DOI:** 10.1155/2013/609727

**Published:** 2013-02-20

**Authors:** Maxwel Capsy Boga Ribeiro, Luiz Roberto Lopes, João de Souza Coelho Neto, Valdir Tercioti, Nelson Adami Andreollo

**Affiliations:** Department of Surgery, Digestive Diseases Surgical Unit, Faculty of Medical Sciences, State University of Campinas (UNICAMP), Rua Tessália Vieira de Camargo, 126 Cidade Universitária Zeferino Vaz, 13083-887 Campinas, SP, Brazil

## Abstract

Gastric adenocarcinoma after gastric bypass for morbid obesity is rare but has been described. The diet restriction, weight loss, and difficult assessment of the bypassed stomach, after this procedure, hinder and delay its diagnosis. We present a 52-year-old man who underwent Roux-en-Y gastric bypass 2 years ago and whose previous upper digestive endoscopy was considered normal. He presented with weight loss, attributed to the procedure, and progressive dysphagia. Upper digestive endoscopy revealed stenosing tumor in gastric pouch whose biopsy showed diffuse-type gastric adenocarcinoma. He underwent total gastrectomy, left lobectomy, distal pancreatectomy and splenectomy, segmental colectomy, and bowel resection with esophagojejunal anastomosis. The histopathological analysis confirmed the presence of gastric cancer. The pathogenesis of gastric pouch adenocarcinoma is discussed with a literature review.

## 1. Introduction 

Obesity is a global problem, causing significant morbidity and mortality [1]. Surgery is the only treatment in morbidly obese patients causing effective weight loss and beneficial long-term results. Postoperative outcome assessments have clearly demonstrated the positive effect of bariatric surgery on weight loss and subsequent improvement or resolution of type 2 diabetes, obstructive sleep apnea, hypertension, and dyslipidemias [1–3]. The original gastric bypass procedure was performed in 1966 by Mason and Ito [4]. The Roux-en-Y gastric bypass (RYGBP) is the most common procedure currently performed for surgical treatment of morbid obesity. Long-term complications may occur after bariatric surgery but cancer is rare [5]. We report a case of gastric adenocarcinoma after RYGBP and a review of the literature.

## 2. Case Report 

**Figure 1:**
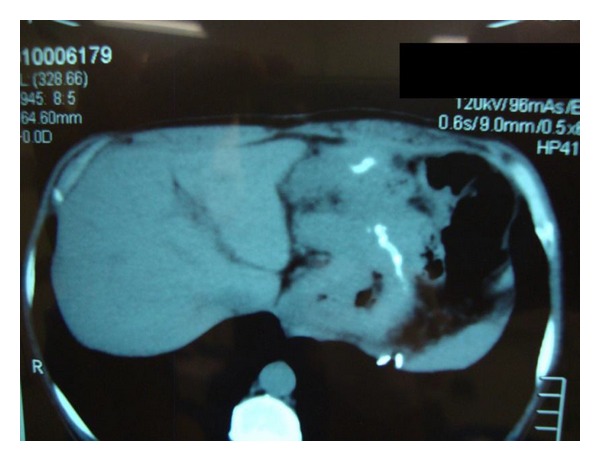
CT scan showing the lesion affecting stomach and with invasion of the left hepatic lobe and the pancreas.

A 52-year-old man presented with severe dysphagia 1 month ago. On the examination, only pallor was noted. The laboratory findings were normal, except for a microcytic anemia with hemoglobin 8.9 g/dL. He had undergone RYGBP for the treatment of morbid obesity 2 years ago, with Body Mass Index (BMI) of 42 and type 2 diabetes. An upper digestive endoscopy performed before surgery was considered normal and revealed *Helicobacter pylori *(Hp) positive. He treated the Hp using amoxicillin and clarithromycin and the endoscopy was repeated showing Hp negative. After surgery he had dietary restriction and lost 40 kg. Upper digestive endoscopy revealed a stricture lesion in the gastric pouch and the biopsies confirmed a signet-cell undifferentiated adenocarcinoma. The computed tomography (CT scan) revealed a large abdominal tumor affecting the excluded stomach and with signs of invasion of the left hepatic lobe and the pancreas (Figure 1).

**Figure 2:**
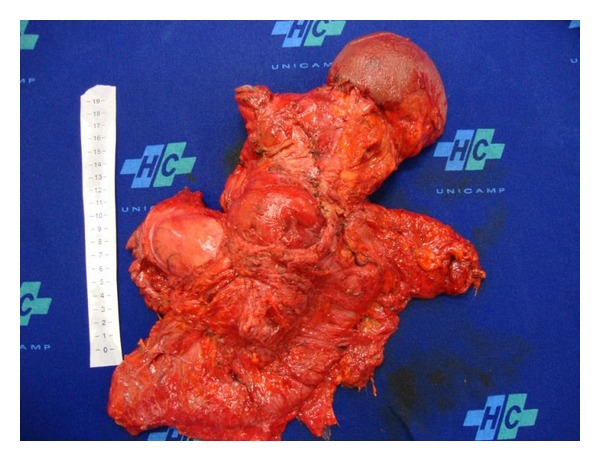
Surgical specimen.

**Figure 3:**
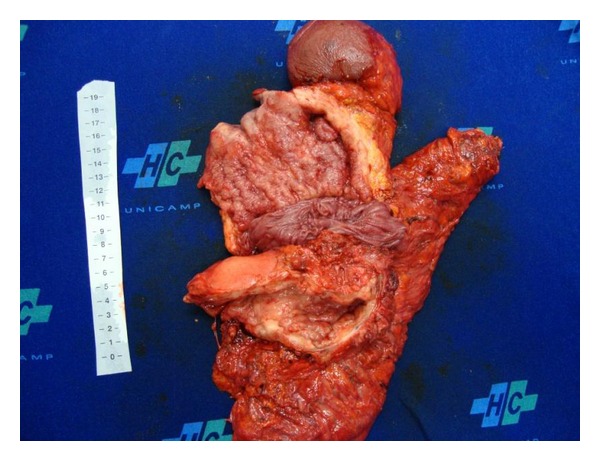
Detail of the tumor: gastric pouch and bypassed stomach sectioned.

The patient underwent total gastrectomy, including the excluded stomach, distal pancreatectomy and splenectomy, left hepatectomy and segmental colectomy with Roux-en-Y esophagojejunal anastomosis, and primary colocolic anastomosis because there was true carcinomatosis invasion of adjacent organs (Figures 2 and 3). Twenty-five lymph-nodes were resected and fourteen had metastasis. He had an uneventful postoperative period. Chemotherapy was performed during six months with good tolerance. 

**Figure 4:**
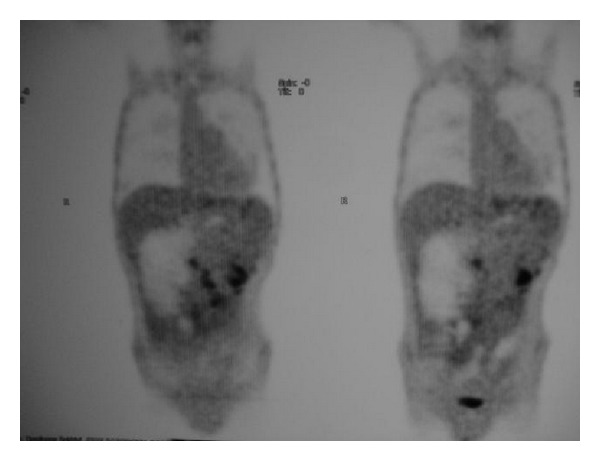
PET-CT showed peritoneal carcinomatosis in coronal reconstruction.

**Figure 5:**
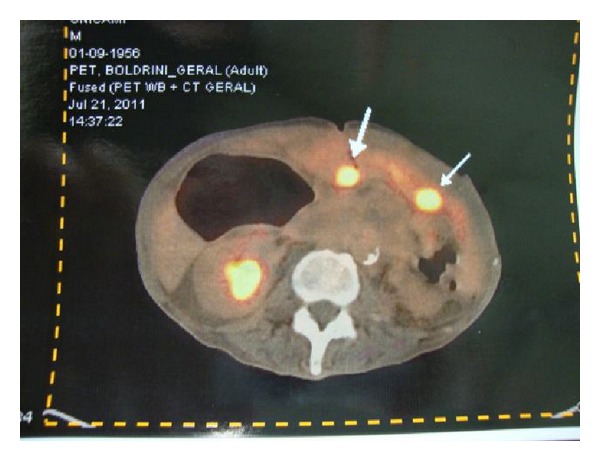
Peritoneal lesions with high SUV on PET-CT.

After 16 months, the patient was admitted to the emergency with intestinal obstruction. Exploratory laparotomy revealed numerous intestinal adhesions and bowel obstruction at multiple points. He underwent an ileocolic anastomosis. Biopsies of the adhesions were inconclusive. Nevertheless, a PET-CT showed peritoneal carcinomatosis (Figures 4 and 5). Palliative chemotherapy was again introduced. His condition deteriorated, and he died after five months. 

## 3. Discussion 

The incidence of esophagogastric cancer after bariatric surgery is rare; however, about 30 cases of adenocarcinoma have been described in the last years [2, 5]. The etiology has not been clearly elucidated, and possible factors could be chronic reflux, stasis of food and acid in the pouch and lower esophagus causing chronic mucosal irritation, and ischemic damage due to the band when it is present [5, 6]. Retrograde gastroscopies of the bypassed stomach after RYGBP (using a pediatric colonoscope) found an 87% incidence of macroscopic gastritis, with microscopic confirmation in 45% of the cases and with progression to intestinal metaplasia in 10% [7–9].

Endoscopy is considered the most sensitive diagnostic modality to aid in the detection of premalignant dysplasia or malignant gastric lesions [10]. Endoscopic access to the excluded stomach might be possible through the afferent limb of the previously loop gastrojejunostomy or through the Roux limb, although this is technically challenging and can be unreliable [11]. With a routine upper gastrointestinal study, oral contrast is unlikely to reflux up the biliary limb to reach the gastric remnant [7, 9, 12]. CT scanning is and should remain the primary screening modality, where small or early lesions might not be detected [11]. Percutaneous puncture under ultrasound control is possible [13]. Finally, if an endoscopic or radiologic diagnosis cannot be made, access to the excluded stomach should be the next step in the evaluation [14]. 

There are conditions that have been associated to greater risk of gastric adenocarcinoma, such as family history, blood type-A, hereditary nonpolyposis colon cancer, and the Li-Fraumeni syndrome. There also exist precancerous lesions such as adenomatous polyps, dysplasia, intestinal metaplasia, and Menetrier's disease. In patients with these conditions with higher risk of developing gastric cancer, a resection of the bypassed stomach can be considered at the time of RYGBP [15].

The diagnosis of esophagogastric malignancies after bariatric procedures can be difficult because weight loss, vomiting, and inability to eat normal quantities of food can be attributed to the expected results of surgery [2, 5].

The cases of gastric carcinomas after bariatric surgeries previously reported by Raijman et al. [13], Lord et al. [16], Khitin et al. [17], Corsini et al. [18], Escalona et al. [15], Watkins et al. [11], and Harper et al. [19] presented a variety of symptoms, but abdominal pain was more frequent, and the usual evaluation methods were unsuccessful in providing a diagnosis. All patients reported were diagnosed with adenocarcinoma in the excluded stomach after laparotomy; therefore, this a difficult diagnosis. In our case, preoperative upper digestive endoscopy was considered normal for 2 years, and after the surgery endoscopy diagnosed the disease, because the tumor invaded the gastric pouch. The same situation was described by Trincado et al. 5 years after gastric bypass [20]. 

On the other hand, gastric adenocarcinomas have been described after vertical banded gastroplasty (VBG). Ziraik et al. [21], Papakonstantinou et al. [22], Belhaj et al. [23], Jain et al. [6], and Chebib et al. [24], respectively, described gastric adenocarcinomas in patients 2 years, 6 years, 10 years, 15 years, and 18 years after VBG. These authors suggest that these patients should be monitored periodically by endoscopy after operation. 

The *Helicobacter pylori *(Hp) is one of the etiologic factors associated to gastric cancer [25, 26]. Thus, due to the presence of this bacteria in obese population undergoing Roux-en-Y bypass gastric surgery and the concern that it may exacerbate postoperative foregut symptoms and increase gastric cancer risk, this led the surgeons to adopt a policy of Hp systematic eradication preoperatively. Cerqueira et al. suggested that the 14-day triple therapy (proton pump inhibitor, clarithromycin, and amoxicillin) is more effective than 7 days [27].

The authors, in recent publications, advise that an adequate preoperative gastric evaluation by upper digestive endoscopy should be performed on all candidates before surgical treatment for obesity [28]. 

Finally, there must be a high index of suspicion, if any sign of malignancy was found during an endoscopy, for earlier detection and treatment of gastric carcinoma, improving patient outcomes.
